# SG-APSIC1059: Impact of a single intervention as part of a antimicrobial stewardship in a surgical unit of a tertiary-care referral center for neurosurgery

**DOI:** 10.1017/ash.2023.4

**Published:** 2023-03-16

**Authors:** Kavita Raja, Dinoop Korol Ponnambath, Shiny Biju, Jyothi Embekkat Kaviyil

**Affiliations:** Sree Chitra Tirunal Institute for Medical Sciences and Technology, India; Trivandrum, Sree Chitra Tirunal Institute for Medical Sciences and Technology, India; Trivandrum, Sree Chitra Tirunal Institute for Medical Sciences and Technology, India; Trivandrum, Sree Chitra Tirunal Institute for Medical Sciences and Technology, India

## Abstract

**Background:** Antimicrobial resistance is a worldwide problem leading to increasing deaths due to intractable infections, especially in postoperative patients who have extended stays in ICUs due to other surgical complications. Carbapenem and colistin resistance has been increasing here; hence, it was decided to monitor and control antibiotic use. In the neurosurgery unit of a quaternary-care hospital in South India, surgical prophylaxis was chosen as less problematic area in which to implement antibiotic stewardship. **Objective:** To study the difference in the antibiogram pattern of isolates from neurosurgery postoperative patients, before and after the introduction of a surgical antimicrobial prophylaxis policy from the UK National Health Service (NHS). **Methods:** After the implementation of a new surgical prophylaxis protocol taken from the UK NHS guidelines, we studied its impact by analyzing the antibiogram before implementation (period 1 from January 1, 2020, to December 31, 2020) and after implementation (period 2 from April 1, 2021, to September 30, 2021). This period corresponded to the same number of isolates as the earlier period. **Antibiogram criteria:** All clinically relevant infections due to the ESKAPE pathogens were included in the antibiogram. The antibiotics analyzed included β-lactams, cephalosporins, β-lactam- lactamase combinations, carbapenems, aminoglycosides, colistin and tigecycline for gram-negative bacilli and penicillin, oxacillin, aminoglycosides, vancomycin, and linezolid for gram-positive cocci. For analysis, the difference was deemed significant according to the criteria stated in CLSI document M39-A4 (4th edition, January 2014). **Results:** In period 1, 170 isolates were tested, and in period 2, 162 isolates were tested. Among the isolates, *Enterococcus* spp and *Enterobacter* spp were too few in number for a comparison. For the gram-negative bacilli, *E. coli*, *Klebsiella pneumoniae*, and *Acinetobacter baumannii*, the differences were significant for the β-lactam–lactamase combinations, carbapenems, and amikacin, with higher susceptibility in period 2. For *Staphylococcus aureus*, oxacillin, erythromycin, and clindamycin showed a significant increase in susceptibility in period 2. Relevant tables and a graph will be included in the presentation with detailed discussion. **Conclusions:** Controlled surgical prophylaxis strictly implemented can lead to a significant change in the antibiotic susceptibility pattern among isolates causing healthcare-associated infections among postoperative patients in intensive care units.

